# The Role of Bacterial and Fungal Superinfection in Critical COVID-19

**DOI:** 10.3390/v14122785

**Published:** 2022-12-14

**Authors:** Tamara Seitz, Johannes Holbik, Alexander Grieb, Mario Karolyi, Julian Hind, Georg Gibas, Stephanie Neuhold, Alexander Zoufaly, Christoph Wenisch

**Affiliations:** 1 Department of Infectious Diseases and Tropical Medicine, Klinik Favoriten, 1100 Vienna, Austria; 2 Faculty of Medicine, Sigmund Freud University, 1020 Vienna, Austria

**Keywords:** COVID-19, SARS-CoV-2, fungal infection, superinfection, candidemia, CAPA

## Abstract

Background: The range of reported rates of bacterial and fungal superinfections in patients with a severe course of COVID-19 is wide, suggesting a lack of standardised reporting. Methods: The rates of bacterial and fungal superinfection were assessed using predefined criteria to differentiate between infection and contamination. Results: Overall, 117 patients admitted to the Intensive Care Unit due to severe COVID-19 were included. Overall, 55% of patients developed a superinfection and 13.6% developed a fungal superinfection (5.9% candidemia and 7.7% CAPA). The rate of ventilator-associated pneumonia was 65.2%. If superinfection was detected, the length of hospital stay was significantly longer and the mortality was especially increased if candidemia was detected. An increased risk of superinfection was observed in patients with pre-existing diabetes mellitus or chronic heart failure. The presence of immunomodulating therapy did not seem to have an impact on the frequency of superinfections. Conclusion: Increased awareness of high superinfection rates, fungal infections in particular, in patients suffering from severe COVID-19 is necessary.

## 1. Introduction

Despite a considerable and continuously growing body of evidence regarding the clinical course of coronavirus disease 19 (COVID-19) caused by severe acute respiratory syndrome coronavirus 2 (SARS-CoV-2), unanswered questions regarding the role of bacterial and fungal superinfections in patients with a severe course remain. The reported rate of those superinfections ranged from 1% to 86.6% in critically ill patients suffering from COVID-19 in recent European, Asian and American studies [1,2,3], suggesting a lack of standardised reporting. The risk of ventilator-associated pneumonia (VAP) in patients with COVID-19 is estimated to be 31% to 44% [4,5].

Chen et al. [3] reported that 71% of the patients received antibiotics and 15% received antifungal therapy, while a pathogen was only detected in 5% of the patients. The increased use of antimicrobial agents during the COVID-19 pandemic has been viewed critically [6], and may have resulted in a reduced detection rate of pathogens in culture [7], as culture-independent methods are likely underutilised. Recent studies showed that patients with severe COVID-19 had a high risk of development of an invasive fungal infection (e.g., candidiasis, aspergillosis, mucormycosis, and histoplasmosis), which are especially difficult to detect using traditional methods [8]. Invasive candidiasis (IC) occurs at a significantly higher rate in patients admitted to an Intensive Care Unit (ICU) with COVID-19 compared to patients admitted to ICU without COVID-19 [9].

COVID-19 associated pulmonary aspergillosis (CAPA) is a further severe complication of COVID-19. A meta-analysis found incidence rates between 19.6% and 33.3% in critically ill patients with COVID-19 [10], although several methodological limitations, including small sample sizes, retrospective designs and variable distinction between invasive infection and contamination, must be considered.

Both lack of recognition and misdiagnosis of superinfection can lead to negative health outcomes. Inappropriate antimicrobial therapy actively harms patients and contributes to antimicrobial resistance [11]. Furthermore, COVID-19 patients with superinfections were shown to have a significantly higher mortality [2,12,13,14], making rapid and accurate diagnosis and initiation of appropriate therapy crucial.

An adequate and qualitative evaluation of the rate of superinfection in patients with COVID-19 is urgently required. Therefore, an investigation of fungal and bacterial superinfection in patients with severe COVID-19 in the ICU was performed to evaluate clinical and microbiological findings.

### Aims

The main aim of this study was to define the number of true fungal and bacterial superinfections in patients with severe COVID-19 and to describe their characteristics (e.g., time of development or microbiology). A differentiation between infection and contamination was made by infectious-disease physicians using predefined criteria. A secondary aim was to evaluate the impact of superinfections on the clinical outcome of the patient. Furthermore, risk factors for developing superinfections in critically ill patients with severe COVID-19 were evaluated.

## 2. Methods

A retrospective analysis of all patients admitted to the ICU of the Department of Infectious Diseases and Tropical Medicine, Clinic Favoriten, Vienna, from January to June 2021, was performed. During this time frame, only critically ill patients with a SARS-CoV-2 infection confirmed by PCR were admitted to this unit. The study was approved by the local ethics committee (EK 20-790-VK). Baseline patient characteristics, outcome parameters and microbiological data were collected until death or discharge from hospital. Microbiological data was evaluated and allocated:

(1) Contamination.
(2) Blood stream infection (BSI), including catheter-related blood stream infection (CRBSI) and IC (invasive candidiasis).
(3) Bacterial pneumonia, subdivided into community-acquired pneumonia (CAP), hospital-acquired pneumonia (HAP) and ventilator-associated pneumonia (VAP).
(4) COVID-19-associated pulmonary aspergillosis (CAPA), subdivided into highly likely and likely CAPA.

Points 2–4 were regarded as superinfections and were diagnosed with the following criteria:

### 2.1. Blood Stream Infections

BSI is considered if a bacterium or fungus is detected through T2MR [15] or growth in blood culture. If the detected pathogen is isolated in a different sample type, e.g., a urine sample, and/or at least two different specialists in infectious diseases retrospectively agree that the detected pathogen reflects the focus of infection and fits the nature of the underlying infection, BSI is regarded as infection and not contamination.

Following the guidelines of IDSA [16], diagnosis of CRBSI is made if

- A colony count of microbes grown from blood obtained through the catheter hub;
- is at least 3-fold greater than the colony count from blood obtained from a peripheral vein
- OR
- Growth in microbes from a blood sample drawn from a catheter hub is detected at least 2 h before microbial growth in a blood sample obtained from a peripheral vein

### 2.2. Bacterial Pneumonia

Following the pneumonia definition of ECDC [13], a combination of radiological, clinical and microbiological criteria was necessary to diagnose pneumonia:

1. Radiological
  - New or worsening infiltrates on Chest X-Rax or CT Thorax.
2. Clinical
  - Temperature > 38 °C without other cause.
    and/or
  - Leukopenia (<4000 WBC/mm^3^) or leucocytosis (>12,000 WBC/mm^3^)
    and at least one of the following:
  - New onset of purulent sputum or change in characteristics.
  - Suggestive auscultation.
  - Worsening gas exchange.
3. Microbiological
  - Positive culture of sputum, tracheal aspirates or bronchoalveolar lavage (BAL) with a threshold ≥10^4^ CFU/mL.
    Or
  - Positive qualitative result in RT-PCR of tracheal aspirates or bronchoalveolar lavage (BAL).

Pneumonia was defined as CAP when symptom onset was within 48 h of hospitalization in non-intubated patients. HAP was defined as symptom onset ≥48 h after hospitalization [17]. Pneumonia was categorized as VAP when occurring at least 48 h after initiation of mechanical ventilation [17].

### 2.3. CAPA

Relying on the criteria of Armstrong James et al. [18], prerequisite for the diagnosis of CAPA is respiratory deterioration or persistence of poor respiratory function with no other sufficient explanation or progression of pathology on X-ray or CT scan. Depending on available microbiological tests, patients are sorted in groups of highly likely CAPA, likely CAPA and unlikely CAPA.

### 2.4. Statistical Analysis

Baseline parameters (age, gender, co-morbidities, days between symptom onset and ICU admission), present therapy and outcome parameters of all included patients were collected.

Continuous variables were summarized as mean and standard deviation (SD) and the groups were compared using the *t*-test (with Welch correction). Categorical variables between groups were compared using the Fisher exact tests. A *p*-value < 0.05 was assumed as statistically significant. The calculations were performed with Graph Pad Prism (GraphPad Prism version 9.3.0 for macOS, GraphPad Software, San Diego, CA, USA, www.graphpad.com).

## 3. Results

At total of 117 patients were included in the analysis. The patient characteristics and therapies received are listed in Table 1.

The outcome parameters of the included patients are listed in Table 2.

### 3.1. Rate of Superinfections

In 54.7% (64 of 117) of patients with severe COVID-19 requiring ICU admission, at least one superinfection was diagnosed.

#### 3.1.1. Bacterial Pneumonia

**Figure 1:**
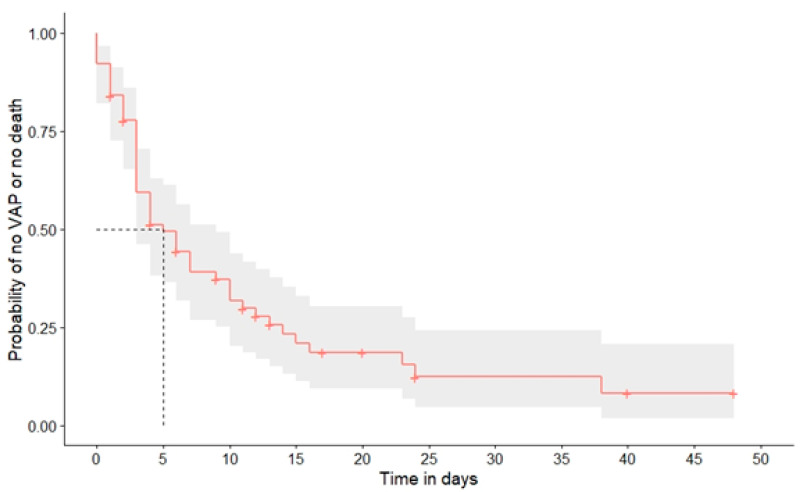
Risk of developing VAP (ventilator-associated pneumonia) or dying after intubation.

The rate of bacterial pneumonia was 42.7% (five cases defined as HAP and 45 as VAP). There were no cases of CAP in this cohort. The rate of detected HAP was 10.4%. Given that 69 patients were intubated and mechanically ventilated at time of diagnosis, the rate of detected VAP in ventilated patients was 65.2%. The risk of developing VAP during the course of hospital stay is shown in Figure 1.

#### 3.1.2. CAPA

The rate of diagnosed CAPA was 7.7% (9 out of 117) in this cohort. Five cases were defined as highly likely and four as likely CAPA.

Detailed information about HAP, VAP and CAPA are shown in Table 3.

#### 3.1.3. Blood-Stream Infections

In 30.77% (36 out of 117) of patients, a pathogen was detected in BC (blood culture) and/or T2MR. 16.2% (19 out of 117) were defined as true bloodstream infections, 9 (7.7%) were judged to be as CRBSI (catheter-related bloodstream infections) and 6% were cases of IC. Detailed data is shown in Table 4.

#### 3.1.4. Influence of Superinfections on Clinical Outcome

The 28-day mortality was 15.6% in patients without superinfection compared to 28.3% in patients with superinfection—the difference was not statistically significant (*p* = 0.115).

**Figure 2:**
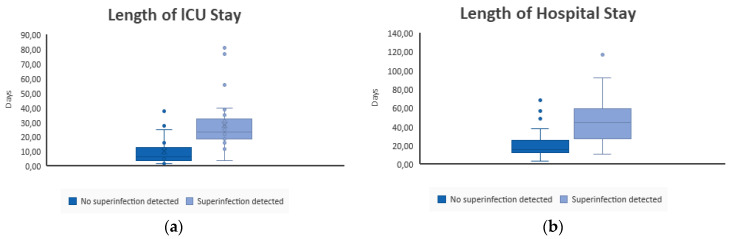
(**a**) Length of ICU stay (in days) of patients with no detected superinfection and with detected superinfection. (**b**) Length of hospital stay in general (in days) of patients with no detected superinfection and with detected superinfection.

In Figure 2a,b, the mean length of stay at an ICU and hospital in general in patients with and without detected superinfections is shown. The difference is significant (*p* < 0.001).

The influence on the presence of BSI and IC is seen in Table 5; the influence on the presence of VAP or CAPA in ventilated patients in Table 6.

#### 3.1.5. Risk Factors of Superinfection

Pre-existing diabetes mellitus (*p* = 0.031) and chronic heart failure (*p* = 0.017) are significantly associated with a higher risk of superinfection. Sex assigned at birth, other comorbidities (chronic heart failure, chronic renal failure, chronic lung diseases, hypertension, thyroid disorder, active cancer and obesity) as well as immunomodulating therapy do not have a significant impact.

#### 3.1.6. Blood-Stream Infections

Neither pre-existing co-morbidities nor any kind of immunomodulating therapy nor sex or age have a significant impact on rate of BSI in general. Of note, regarding IC, is that a higher age (*p* = 0.001) and pre-existing diabetes mellitus (*p* = 0.04) have been found to be risk factors.

#### 3.1.7. Bacterial Pneumonia

Pre-existing diabetes mellitus (*p* = 0.004) is significantly associated with a higher risk of HAP. Sex assigned at birth, other comorbidities as well as immunomodulating therapy do not have a significant impact. In ventilated patients, neither pre-existing co-morbidities or any kind of immunomodulating therapy nor sex or age have a significant impact on rate of VAP.

#### 3.1.8. CAPA

Neither pre-existing co-morbidities nor any kind of immunomodulating therapy nor sex or age were found to have a significant impact in rate of CAPA.

## 4. Discussion

This study cohort demonstrated a higher rate of superinfection in comparison to other published data [19,20,21]. Fifty-five percent of the patients in our cohort developed a superinfection, 13.6% developed a fungal superinfection, of which 5.9% were candidemia and 7.7% were CAPA. While a high rate of these infections, especially fungal superinfections, can in part be explained by the baseline characteristics, including comorbidities, severe course of disease, mechanical ventilation and immunosuppressive drugs, a higher rate of superinfection detection might also have contributed to these results. In a specialized infectious disease unit, clinical suspicion may be higher and culture-independent methods such as T2MR or PCR are more easily accessible, as those are performed directly on-site by the treating physicians. These methods may be more suitable for rapid and accurate diagnosis, as culture-dependent methods commonly yield false-negative results, especially in patients with invasive fungal infections. It is, therefore, possible that these results more closely reflect the true rate of fungal superinfection in this patient cohort. The presence of superinfection was significantly associated with a longer length of stay in the ICU and hospital in general; a trend in increased mortality could be observed.

### 4.1. Fungal Infections

Our data urges the need to screen for invasive candidemia in patients with severe COVID-19, not only due to the high rate of nearly 6% but also because of the significantly increased mortality and length of hospital stay. The high mortality and the higher incidence of IC in patients with COVID-19 compared to non-COVID-19 patients were shown in other studies as well [22,23]. Advanced age and presence of diabetes mellitus were associated with a higher risk of the development of IC in our study, while immunosuppressive therapy was not, replicating published data [24]. A previous multivariate logistic regression analysis demonstrated that advanced age is a further independent risk factor for mortality in COVID-19 patients with IC [22]. Zhou et al. postulated that the age-related decrease in immunological functions may result in a deficiency in controlling SARS-CoV-2 replication and fungal superinfection [8].

Diagnosis of IC is often challenging given that the “gold standard” of the blood culture has been shown to have a low sensitivity of 21–71% for Candida species [25]. To improve the diagnosis of candidaemia, blood cultures can be combined with other methods. Tests assessing the presence and targeting of fungal antigens or antibodies are commonly used for the diagnosis of invasive fungal diseases. For example, the cell walls of most pathogenic fungi contain Β-D-glucan (BDG) which can, thus, be used as a surrogate marker for fungal infections. A problem associated with this test is that it is not specific for IC and the high rate of false-positive results. Positive results should, therefore, always be confirmed with another method. Circulating levels of mannan or antibodies (CandidaAg) directed against this component of yeast cell walls can be used to detect invasive candidiasis in patients. However, the validity of this method is hampered by the quick clearance of mannan from the serum. In a recent retrospective study [26], an insufficient sensitivity of 52–65% and high specificity of 98% was demonstrated. T2MR [15] is a promising method utilizing T2 Magnetic Resonance for the rapid detection of five different Candida species in whole blood samples. Recent studies demonstrated that T2MR might be superior to blood culture [27,28].

Although the rate of 7.7% of diagnosed CAPA in our cohort is high, it is still lower than in other published studies [10,29]. Since we used defined criteria to diagnose CAPA unlike other published studies to prevent assessment of contamination as infection, it can be assumed that these results more closely reflect the true rate. In our cohort, the median stay at hospital was 36.5 days if no CAPA was diagnosed compared to 90 days if CAPA was evaluated as very likely. Therefore, here again, rapid diagnosis and treatment is essential.

### 4.2. Bacterial Pneumonia

A further concern is the high rate of bacterial pneumonia in this cohort (42.7% of all included patients), especially if mechanical ventilation was needed (65.2% of all ventilated patients). In a third of the detected VAPs, the infection was polymicrobial. The most commonly detected pathogen was Staphylococcus aureus, at a mean time of 5–6 days after intubation. Several publications have already noted the increased incidence of VAP in patients with severe COVID-19 in comparison to non-COVID-19 ICU patients [30,31]. While publications before the pandemic reported rates of VAP between 10 and 33% [29], a recent review found that 30 to 60% of COVID patients requiring respiratory support develop VAP [32].

### 4.3. Pathogenesis of Bacterial and Fungal Superinfection

On the one hand, discussed reasons for the high rate of bacterial or fungal infections are the required intensive care including, e.g., mechanical ventilation, broad spectrum antimicrobial therapy and parenteral nutrition, which are all known risk factors for the development of superinfections [33,34,35]. On the other hand, recent studies have shown that patients suffering from COVID-19 present with a reduced number of CD4 and CD8 cells [35], resulting in a declined adaptive form of immunity. It can be assumed that these factors establish a favourable environment for bacterial and especially fungal superinfections [36,37,38]. Additionally, excessive production of both anti-inflammatory cytokines, such as IL4 and IL10, and pro-inflammatory cytokines, such as IL6, IL2, and TNFα, have been shown to favour lung damage and the exhaustion of the immune system [21]. Further, a topic of discussion is altered lung or intestinal microbiota due to SARS-CoV-2 effecting the immune system, which could benefit the development of superinfections [8,39]. Moreover, severe SARS-CoV-2 pneumonia results in an inflamed alveolar space which provides an ideal environment for microbial growth [40]. Regardless, a lot is still unknown and further research is necessary to better understand the pathogeneses of superinfections in COVID-19 patients.

### 4.4. Risk Factors for the Development of Superinfection

In this cohort, an increased risk of superinfection was observed in patients with pre-existing diabetes mellitus or chronic heart failure. An explanation might be that persons suffering from diabetes mellitus often have a compromised innate immunity favouring infections [8,41]. Previous studies demonstrated that diabetes mellitus and cardiovascular disease were associated with severity of the disease, but the authors failed to evaluate the presence of superinfection [42,43]. A reason for the increased severity might be the higher risk of superinfection as shown in our cohort, once again demonstrating the need for rapid diagnosis and treatment. Immunosuppressive therapy was not associated with a higher rate of superinfection.

### 4.5. Limitations and Strength

We acknowledge that this study has some limitations. Firstly, it has a retrospective design and is a single-centre study. Secondly, the sample size is too small for some of the detected pathogens or infections to achieve significant differences. However, the strength of the study is that predefined criteria were used to differentiate between infection and contamination. The study was performed at a specialized department for infectious diseases resulting in advanced knowledge and diagnostic possibilities using non-culture-dependent methods. It must be taken into consideration that such diagnostic methods are not available in every department, which may result in worse clinical outcome than described in this cohort should superinfection truly be present.

## 5. Conclusions

To summarize, a high rate of bacterial and fungal superinfections is present in patients suffering with severe COVID-19. Screening for fungal infection, in particular, is essential for early diagnosis and treatment to improve patient outcomes.

## Figures and Tables

**Table 1:** Basic parameters and therapy of all 117 included patients. * Administrated as part of a clinical study.

Basis Parameters	
Mean age (years ± SD)	57.2 (±11.9)
Female sex (%)	45 (38.5%)
Co-morbidities	
Arterial hypertension	81 (58.3%)
Obesity (BMI 30–40)	57 (41%)
Severe obesity (BMI > 40)	18 (12.9%)
Diabetes mellitus II	48 (35.3%)
Chronic lung disease	27 (19.4%)
Hypo/hyperthyroidism	24 (17.3%)
Chronic arterial disease	19 (13.7%)
Chronic renal failure	9 (6.5%)
Chronic heart failure	7 (5%)
Active cancer	5 (3.6%)
Immunosuppression	4 (2.9%)
Days between symptom onset and ICU admission (± SD)	9.88 (± 6.9)
Therapy	
Immunomodulating	
Dexamethason	117 (100%)
Tocilizumab	12 (8.6%)
Asunercept *	1 (0.7%)
Antiviral therapy	
Remdesivir	51 (43.6%)
Camostat	3 (2.6%)
Lopinavir/Ritonavir	3 (2.6%)
Chloroquin/hydroxychloroquin	1 (0.9%)
Parenteral nutrition	100 (73.5%)

**Table 2:** Outcome parameters of all 117 included patients.

Outcome Parameters	
Invasive ventilation (%)	69 (59%)
Length of invasive ventilation (days ± SD)	15 (±10.4)
Tracheotomy (%)	34 (24.5%)
ECMO support (%)	11 (7.9%)
Central venous catheter (%)	109 (80.7%)
Catecholamine support (%)	86 (63.2%)
Continuous renal replacement therapy (%)	10 (7.2%)
Length of ICU stay (days ± SD)	27.3 (±16.14)
Length of hospital stay (days ± SD)	45.6 (±23.23)
28-day mortality (%)	25 (21.4%)
Clinical status at day 28 after ICU admission	
Discharged	48 (41.03%)
Normal ward	21 (17.95%)
Still at ICU	23 (19.66%)
Dead	25 (21.4%)

**Table 3:** Clinical data and pathogens of HAP (hospital-acquired pneumonia), VAP (ventilator-associated pneumonia and CAPA (COVID-19-associated pulmonary aspergillosis).

	HAP (n = 5)	VAP (n = 45)	CAPA (n = 9)
Median time since detection (+/−SD) in days			
Since symptom onset of COVID-19 infection	15 (10.14)	18 (10.69)	21 (10.64)
Since hospital admission	7 (7.4)	8.5 (8.05)	13.69 (8.67)
Since ICU admission	5 (4.5)	5 (4.75)	10.6 (5.6)
Since intubation	N/A	5.5 (4.24)	8.15 (6.57)
Detected pathogens		17.7% *S. aureus* 15.6% *H. influenzae* 11.1% *K. pneumoniae* 8.8% *P. aeruginosa* 6.7% *S. maltophilia* 4.4% *E. coli* 2.2% *M. catarrhalis* 33.5% *polymicrobial*	
	25% MRSA		44.4% *A. fumigatus*
	25% *P. aeruginosa*		11.1% *A. niger*
	50% *polymicrobial*		44.4% unknown

**Table 4:** Clinical data and pathogens of BSI (blood-stream infection), including CRBSI (catheter-related blood-stream infection) and IC (invasive candidiasis).

	All BSI (n = 19)	CRBSI (n = 9)	IC (n = 7)
Median time since detection (+/−SD) in days			
Since symptom onset	19 (6.33)	21 (5.62)	27 (4.61)
Since hospital admission	12 (7.16)	13 (4.8)	14 (4.95)
Since ICU admission	9 (5.41)	10 (4.57)	10 (6.87)
Detected pathogens	21% *C. albicans*	33.3% *S. aureus* 22.2% *C. albicans* 22.2% *S. epidermidis* 11.1% *E. faecium* 11.1% *polymicrobial*	85.7% *C. albicans*
	21% *S. aureus*		
	16% *E. faecium*		
	10.5% *E. faecalis*		14.3% *C. parapsilosis*
	10.5% *S. epidermidis*		
	12% *polymicrobial*		

**Table 5:** Influence of presence of BSI (blood-stream infection) or IC (invasive candidiasis) in 28-day mortality or length of stay at ICU (intensive care unit) and hospital. ** no *p*-value available due to low number of cases.

	BSI Diagnosed (n = 19)	BSI Not Diagnosed (n = 98)	*p*-Value	IC Diagnosed (n = 7)	IC Not Diagnosed (n = 110)	*p*-Value
28-day mortality						
	36.8%	18.4%	*p* = 0.121	57.1%	19.1%	*p* = 0.037
Total length of stay at ICU (days)						
Mean	28.42	16.03	*p* = 0.046	28.67	17.40	**
Median	27.50	10.50		32.00	12.00	
SD	18.49	15.27		9.45	16.33	
Min-Max	4–77	2–81		18–36	2–81	
Total length of stay at hospital (days)						
Mean	46.00	29.52	*p* = 0.019	46.00	31.29	**
Median	45.00	22.00		46.00	24.00	
SD	16.26	22.81		8.49	22.84	
Min-Max	11–68	4–117		40–52	4–117	

**Table 6:** Influence of presence of VAP (ventilator-associated pneumonia) or CAPA (COVID-19-associated pulmonary aspergillosis) in 28-day mortality or length of stay at ICU (intensive care unit) and hospital in patients requiring invasive ventilation. ** no *p*-value available due to low number of cases.

	VAP Diagnosed (n = 25)	VAP Not Diagnosed (n = 44)	*p*-Value	CAPA Highly Likely (n = 5)	CAPA Likely (n = 4)	CAPA Not Diagnosed (n = 60)	*p*-Value
28-day mortality							
	27.3%	32%	*p* = 0.784	20%	25%	31.7%	**
Total length of stay at ICU (days)							
Mean	27.86	26.25	*p* = 0.765	43.50	30.50	26.08	**
Median	23.00	22.50		43.50	29.50	22.50	
SD	15.41	17.82		17.68	13.38	16.22	
Min-Max	12–81	7–77		31–56	19–44	7–81	
Total length of stay at hospital (days)							
Mean	48.73	38.70		90.00	42.25	42.69	
Median	47.50	39.50		90.00	36.50	42.50	
SD	24.74	18.87		38.18	19.65	19.92	
Min-Max	18–117	13–69		63–117	27–69	13–93	

## Data Availability

Not applicable.
